# Health-related quality of life in patients with venous leg ulcer treated in primary care in Brazil and Portugal

**DOI:** 10.1371/journal.pone.0195990

**Published:** 2018-04-24

**Authors:** Sandra Maria da Solidade Simões de Oliveira Torres, Rhayssa de Oliveira e Araújo, Isabelle Katherinne Fernandes Costa, Manuela Pinto Tibúrcio, Amanda Jessica Gomes de Sousa, Aline Maino Pergola-Marconato, Thalyta Cristina Mansano-Schlosser, Marina de Góes Salvetti, Felismina Rosa Parreira Mendes, Gilson de Vasconcelos Torres, Eulalia Maria Chaves Maia

**Affiliations:** 1 Health Unit of Igapó, Primary Health Care, Municipal Health Secretary, Natal, Brazil; 2 Emergency Care Unit Maria Nazaré Silva dos Santos, Parnamirim, Brazil; 3 Nursing Department - Federal University of Rio Grande do Norte, Natal, Brazil; 4 Onofre Lopes Universitary Hospital, Natal, Brazil; 5 Nursing Department - Fundação Hermínio Ometto (Uniararas), Araras, Brazil; 6 Nursing Department - Faculdade Anhanguera de Campinas, Campinas, Brazil; 7 Medical-Surgical Nursing Department at School of Nursing University of Sao Paulo, Sao Paulo, Brazil; 8 University of Évora, Center for Research in Sport, Health and Human Development – Évora, Portugal; 9 Psychology Department at Federal University of Rio Grande do Norte, Natal, Brazil; QUT, AUSTRALIA

## Abstract

**Background:**

Venous ulcers constitute an important public health problem as they can cause disability with consequences for multiple dimensions of quality of life.

**Objective:**

To describe the quality of life in patients with venous leg ulcer treated in primary care in two cities from Brazil and Portugal.

**Methods:**

This was a cross-sectional comparative study with a non-probabilistic sample of 171 patients with venous leg ulcers who were treated in primary care in two cities from Brazil and Portugal, namely, Natal and Évora. A form covering sociodemographic and health data and the Medical Outcomes Study 36-Item Short-Form Health Survey were used, and descriptive and inferential analyses were performed.

**Results:**

Significant differences in age and income were observed between the two samples. Patients with venous leg ulcer from Brazil had lower income and were younger than those from Portugal. Quality of life scores were significantly higher in Portugal for the physical aspects, pain, and social functioning, among domains, and for the physical health dimension and total score of QOL.

**Conclusion:**

The quality of life was better in Portugal than in Brazil and the differences between the countries need further investigation.

## Introduction

Venous ulcers (VU) represent a public health problem due to the high prevalence and chronicity. The recurrent profile causes significant physical, psychological, social, cultural and spiritual impacts, with long time intervals between onset and healing and high number of relapses when improperly treated [1].

This type of ulceration results from the dysfunction of the deep venous system and venous hypertension combined with inefficient calf and valvar muscles. This painful chronic wound is one of the multiple clinical manifestations of chronic venous insufficiency (CVI) [2,3].

Among all types of ulcers in the lower limbs, those with venous origin represent 75.0% of cases. They are characterized by chronicity and a long and difficult treatment. Most of the affected people (68.0%) live with the ulcer for approximately two years [4,5]. As the number of chronic wounds has increased due to population aging, there has been a consequent high incidence of chronic diseases such as VU [4]. Thus, a greater demand for treatment is expected, with important implications for health services and outcomes [6].

Primary health care was designed to be an important strategy to intervene in the quality of life in people with VU as it allows the creation of bond between health care professionals and the individual and their families within their social environment [7].

Health care for people with VU is complex and requires the effective participation of a multidisciplinary team in order to be efficient, transcending wound healing. Professionals should be properly trained. Interventions are most effective when they are based on protocols [8], because these may guide the care to achieve satisfactory results. However, protocols have not been consistently used in this population.

Because of the complexity of VU, the quality of life (QOL) in people with VU is often compromised. The injury incurs costs to the affected individual and to society due to several factors, including chronic pain, limited mobility, exudate, odor, anxiety/depression, and changes in family relationships, labor practice and social life [9,10].

Venous ulcers are present in diverse contexts and affect different populations. The sociodemographic characteristics of patients with these injuries, which are influenced by their social context, can impact their quality of life.

Based on the above considerations, a question was raised about the sociodemographic characteristics of people with venous ulcers assisted in the primary health care of Brazil and Portugal and their quality of life.

A unique comparative study [11], published in 2013, showed the differences in sociodemographic, clinical and care aspects among the Brazilian and Portuguese population with VU. The two scenarios had specificities that can impact the effectiveness of the assistance and, consequently, the QOL of patients. Thus, the present study aimed to describe the QOL of people with venous ulcers assisted in primary care in Natal (Brazil) and Évora (Portugal).

## Material and methods

### Study design and site

This is a cross-sectional study conducted in primary care units in Natal, Brazil, and Évora, Portugal. The non-probabilistic sample was composed of 171 patients with VU who received treatment in the primary care network of the two cities. In Brazil, the data collection was carried out from February to September 2014, and in Portugal, from June to November 2011, due to the distinct stages of the research.

Natal is the capital of Rio Grande of Norte state with a population of 877.662 inhabitants in the Metropolitan region in 2016 and Human Development Index 0,763 [12].

Health assistance in Brazil is guided by the Unified Health System (SUS), which was created in 1988 by the Brazilian Federal Constitution and is one of the largest public health systems in the world [12].

According to the Municipal Health Secretariat in Natal, there were 37 family health units and basic health units in the city, all of which were included in this study. Patients and families are monitored through family health agents and there is a bond between families and professionals. In the basic health network, there is no such monitoring.

Évora is a Portuguese city located in the Central sub-region of Alentejo, with Human Development Index 0,831. Four primary health care units were linked to the regional health council of Évora and were members of the National Health System of Portugal. The research was conducted in these four units, which consist in three family units and one basic unit.

### Population and sample

The population of participants was composed of people with VU who were assisted in the primary care units. In Natal, all the eligible people were invited to participate, totaling a population of 101 individuals. In Évora, people were selected according to accessibility during the period of data collection, totaling 70 individuals and the sample sizing process occurred by convenience. Thus, the total research sample consisted of 171 people.

The inclusion criteria were: presence of active acute or chronic, infected or not infected venous ulcers during the study period; singular or multiple venous ulcers; presence of ulcers about medium to large in extension; minimum age of 18 years; and ability to understand the Portuguese language. Exclusion criteria were: patients with completely healed ulcers or ulcers that had a mixed origin. Moreover, patients assisted in family health units in which nurses were on vacation during the collection period were also excluded because the patients were difficult to locate without the help of these nurses.

### Data collection

Data were collected by the researchers at the health units or at home during home visits. Two data collection instruments were used: a structured form for the interviews covering sociodemographic and health characteristics; the health-related quality of life form (HRQOL); and the Medical Outcomes Study 36-Item Short-Form Health Survey (SF-36). Data collection was carried out through interviews conducted by the researchers in a quiet and private environment.

Sociodemographic and health variables included gender, age, marital status, education, income, occupation, chronic diseases, sleep pattern and drinking/smoking. According to international parameters, people aged 60 years or older were considered elderly. Minimum wage was converted to the corresponding currency for each country.

The instrument for collection of sociodemographic and health characteristics was based on a VU protocol and it was validated in Brazil and Portugal in 2010 [8,13].

The SF-36 is a widely used and validated multidimensional questionnaire. It consists of 36 items grouped into eight domains: functioning capacity, physical aspects, pain, general health, vitality, social aspects, emotional aspects and mental health. It additionally contains dimensions of physical health and mental health. The scores for domains vary from zero to 100 and higher scores indicate better evaluation of quality of life [14].

### Data analysis

The data were transferred to a Microsoft Excel 2007 spreadsheet, and after correction, were exported and analyzed in the Statistical Package for Social Science for Windows (SPSS) IBM version 20.0. Descriptive statistics (absolute and relative frequencies, mean, standard deviation) were calculated. Normality of data was tested through the Kolmogorov-Smirnov test; the variables showed a non-normal distribution and, therefore, the percentiles were used and the non-parametric Mann-Whitney U test was used to assess the differences between the average of the domains and dimensions of QOL. The statistical significance level of α = 0.05 was adopted.

### Ethical aspects

The study followed the ethical principles contained in the Helsinki Declaration of the World Medical Association [15] and was in accordance with the Resolution 466/12 of Brazil [16]. The study was submitted and approved by the Research Ethics Committee in Brazil under number 256.512 (CAAE 07556312.0.0000.5537) on April 5, 2013. In Portugal, the approval number 10028 was granted on July 23, 2010.

Before inclusion in the study, the patients were informed about the study objectives, and those who agreed to participate signed the free and informed consent (IC) form.

## Results

The sociodemographic profiles of people with VU who were under treatment in primary care were similar in both countries. The surveyed population was predominantly composed of women (61.4% in Brazil, 84.3% in Portugal), aged over 60 years (36.3% in Brazil, 33.9% in Portugal), married or in stable union (37.4% in Brazil, 23.4% in Portugal), with a low level of education (50.3% in Brazil, 36.8% in Portugal), without a profession or occupation (44.4% in Brazil, 34.5% in Portugal), with chronic diseases (35.7% in Brazil, 27.5% in Portugal), sleeping at least 6 hours a day (48.5% in Brazil, 35.1% in Portugal), and non-smokers and non-consumers of alcohol (47.4% in Brazil, 32.2% in Portugal).

The sociodemographic characteristic that differed significantly between the countries was the per capita income (p = 0.001) and the age (p = 0.003). In Brazil, most people with VU (53.2%) earned up to one minimum wage, and in Portugal, most people with VU (40.4%) earned more than one minimum wage. In both cities, most of the people were 60 years old and older, but in Évora the number of elderly people was much higher, with significant differences in relation to Natal (Table 1).

**Table 1:** Distribution of sociodemographic and health characteristics of people treated in primary care in Brazil/Portugal.

Sociodemographic and health characteristics	Brazil n (%)	Portugal n (%)	Total n (%)	p-value*
**Gender**				
Female	67 (66.3)	49 (70.0)	116 (67.8)	0.614
Male	34 (33.6)	21(30.0)	55 (32.2)	
**Age group**				
65 years and older	47 (27.5)	53 (31.0)	100 (58.5)	<0.001
Up to 65 years	54 (31.6)	17 (9.9)	71 (41.5)	
**Marital status**				
Married/stable union	64 (63.4)	40 (57.2)	104 (60.8)	0.412
Single, widow/widower and divorced	37 (36.6)	30 (42.9)	67 (39.2)	
**Education**				
Up to elementary school	86 (85.1)	63 (90.0)	149 (87.1)	0.351
High school and higher education	15 (14.9)	07 (10.0)	22 (12.9)	
**Profession/occupation**				
Absent	76 (75.2)	59 (84.3)	135 (78.9)	0.154
Present	25 (24.8)	11(15.7)	36 (21.1)	
**Per capita income**				
Up to 1 MW^#^	91(90.0)	01 (0.4)	92 (53.8)	<0.001
More than 1 MW	10 (10.0)	69 (98.6)	79 (46.2)	
**Associated chronic diseases**				
Present	61 (60.4)	47 (60.0)	108 (63.2)	0.368
Absent	40 (39.6)	23 (40.0)	63 (36.8)	
**Sleep**				
More than 6 hours per day	83 (82.2)	60 (85.7)	14 (83.6)	0.539
Up to 6 hours per day	18 (17.8)	10 (14.3)	28 (16.4)	
**Alcohol comsuption/smoking**				
Absent	81(80.2)	55 (78.6)	136 (79.5)	0.795
Present	20 (19.8)	15 (21.4)	35 (20.5)	
**Total**	101(59.1)	70(40.9)	171(100.0)	

*Qui-squared test;

^#^MW = minimum wage (R$724.00 in Brazil and €530.00 in Portugal, 2011)

As for the scores on the QOL domains, the physical aspect, pain, social functioning, physical health dimensions and the total score of QOL were significantly higher in Portugal. Except for the general health domain, all other domains had better scores in Portugal than in Brazil.

In Brazil, the domain with the highest score was mental health (median 68.0), while in Portugal was social aspects (median 88.0) (Table 2).

**Table 2:** Differences in the domains and dimensions of the SF-36 between people in Brazil and Portugal.

SF-36	Brazil			Portugal			p-value*
	Percentiles			Percentiles			
Domains	25	50 (median)	75	25	50 (median)	75	
Physical aspects	0.0	0.0	0.0	0.0	12.5	100.0	<0.001
Functional capacity	10.0	30.0	60.0	10.0	30.0	75.0	0.260
Emotional aspects	0.0	33.3	100.0	33.0	67.0	100.0	0.264
Pain	21.0	41.0	62.0	31.0	57.0	84.0	0.016
General Health	35.0	52.0	67.0	30.0	47.0	62.0	0.194
Social aspects	13.0	50.0	75.0	47.0	88.0	100.0	<0.001
Vitality	45.0	65.0	85.0	40.0	65.0	85.0	0.726
Mental health	44.0	68.0	88.0	44.0	76.0	88.0	0.660
Total	31.8	46.1	60.9	41.1	59.9	71.4	0.005
**Dimensions**							
Physical health	25.0	39.0	55.5	31.2	50.5	66.2	0.012
Mental health	38.5	56.0	73.5	45.5	65.0	79.2	0.116

*Mann-Whitney U

## Discussion

Knowing and understanding the socioeconomic profile of patients with VU is important to reflect on the reality they live, because these factors can interfere with their QOL. The sociodemographic profile in this study, both in Brazil and in Portugal, is common in studies of Brazilian people with VU [7,8,17] and of other nationalities [18,19], including Portuguese [20].

The income per capita was one of the items that were significantly higher in Portugal. Low income may be a problem for patients with VU because the treatment requires additional costs in terms of dressings, compression therapy and nutritional adequacy and lifestyle. Although there are policies that implement low-cost treatments, the costs associated with maintaining the treatment of a complex wound, such as VU, are high [21]. Furthermore, frequent follow-up in health institutions is needed, implying transportation-related costs. Because of the specificities of the treatment and clinical manifestations such as pain and impairment of physical mobility, people with UV usually do not exercise paid activity.

A previous comparative study between both countries showed that, in Portugal, patients with VU were older, had better income, and were less frequently employed and presented lesions with more favorable characteristics in the scar healing process than those patients in Brazil [11].

Venous ulcer treatment in a country-specific context should take into account several factors, such as the economy, the type of healthcare system and the local practice of medicine. A comprehensive systematic review of 59 articles that summarized the cost-effectiveness of interventions for complex wound care concluded that the results could be used by decision makers to maximize the deployment of clinically effective and resource efficient wound care interventions. Most of the economic studies included had been carried out in European countries and 16 had been conducted in the United States [21].

Patients with venous ulcer in Brazil were younger than in Portugal. This indicates that Brazilian people developed UV earlier. However, the literature affirms that advanced age is a factor related to the appearance of the venous lesions and to the difficulty of healing [4].

Identifying the sociodemographic characteristics that most interfere with appearance of VU can support the preparation of specific protocols to the demand of each population, achieving better results in the promotion, recovery and rehabilitation of health and, consequently, better QOL.

Concerning the quality of life in people with VU, the mean scores of the domains and dimensions of the SF-36, except for the general health state and vitality domains, proved to be higher in Portugal than in Brazil. This shows that the quality of life in people with VU in Portugal is better than that those in Brazil.

A study of 66 people with VU treated at a Hospital Center in Portugal requested the patients to rate their QOL on a scale from 0 to 10 where 0 corresponded to the worst and 10 to the best possible quality. The survey found a mean of 4.68, corresponding to intermediate values of QOL [20]. This Portuguese study also reported that the social life aspect had better scores and was less impacted by the experiences of the sample. This supports the results of the present study in which social aspects had a higher average in Portugal. Additionally, a small number of patients in the Portuguese study claimed to be unable to have a normal social life, to have difficulty going out and socializing or being with family and friends, to have a desire for isolation and to fear going out to avoid hurting the injury [20].

In a Brazilian study on QOL and chronic venous ulcers using the SF-36 questionnaire, the participants scored were very low means before a compressive intervention. After the intervention, the best result was obtained in general health (mean 38.02, SD 18.44), and the worst in functional capacity (mean 16.10; SD 14.42). The total score averaged only 15.10 (16.42) on a scale with maximum score of 100, where higher scores indicate good results [10].

Another Brazilian study that evaluated musculoskeletal limitations and changes in the quality of life of patients with venous leg ulcers showed a positive and strong correlation (r = 0.773; p = 0.009) between the psychological profile of the SF-36 and social activities. Thus, the presence of venous ulcers in the lower extremities can limit and trigger changes in the quality of life of these patients [22].

In the present study, physical aspects had the lowest average score among QOL domains in both Brazil and Portugal, but averages were significantly lower in Brazil., Pain, the physical health dimension, and the social and physical domains were also significantly worst in Brazil.

Painful leg ulcers, which are associated with restrictions and social isolation, result in a heavy psychosocial burden. The impacts of these and other complications of venous ulcers on overall health and quality of life have only recently been better appreciated. Psychosocial parameters include social isolation, depression, feelings of regret, loss of power, and helplessness [23].

It is noteworthy that the impairment of physical health also affects the social aspects of an individual’s QOL, which was also significantly higher in Portugal. Approximately 20% of people with VU had impaired ambulation; thus, they reported being socially isolated, sleep deprived and emotionally damaged [24]. The limited mobility, pain, excessive exudate, bandages and odor lead to changes in body image, which imply mental and social commitments [24].

It is surprising to note that in some places 95.0% of ulcers heal within 12 weeks, while in other places, the rate is only 20.0% [25]. Thus, the differences in the quality of life of people with UV in the two countries studied here need to be more thoroughly studied in order to find their real determinants, and aid professionals in a better performance of care.

The observed trends in the sociodemographic characteristics, particularly the predominance of young people with low educational level, lower income, and without occupation, has been reinforced in other studies [7,8,17–20] and can indicate worse social conditions influenced by the context of the countries and specificities of the places, with exert influence in the QOL and must be considered in the care.

Because of all the physical and psychosocial limitations that VU cause in daily life, there is an urgent need to adapt health care to the social environment of affected individuals and to boost the pursue for better life conditions. Thus, health professionals need to have a comprehensive perspective prior to treating patients with VU, so as to treat them beyond wound healing, but improving the patients’ QOL.

Nurses have an important role in the healing process, as well as the multi-professional team has, as they assess the patients, plan and perform the treatments, and monitor the patients’ evolution to reduce the impact of the wound [1,7]. The participation of nurses in treatment decision making is essential to improve quality of care [6].

However, this study was limited by the location, as it was only conducted in two cities in different countries, and by the cross-sectional aspect of its design, which prevented the monitoring of respondents to better identify the factors that may have influenced their quality of life. Further studies are needed to identify the interventions and experimental designs in this area.

## Conclusion

The authors concluded that most of the patients with VU in Brazil were adults with per capita income up to one minimum wage while in Portugal the majority was elderly people with per capita income of more than one minimum wage. The other sociodemographic aspects and health characteristics were similar in both countries.

The QOL was better in Portugal than in Brazil, except for the general health domain. Physical aspects of the QOL had the worst scores in both countries. In Brazil, the best result was found for mental health and in Portugal, for social aspects. The differences were significant for physical aspects, pain, social aspects, physical health dimension and total score of the scale. The main difficulties of this study include the low education level of people, as this made the questionnaires difficult to understand. Some unproved assumptions about causality should not be included in the conclusions, based on the available data, and further studies are needed.
